# Light response of gametophyte in *Adiantum flabellulatum*: transcriptome analysis and identification of key genes and pathways

**DOI:** 10.3389/fpls.2023.1222414

**Published:** 2023-09-07

**Authors:** Zeping Cai, Xiaochen Wang, Zhenyu Xie, Zhenyi Wen, Xudong Yu, Shitao Xu, Xinyu Su, Jiajia Luo

**Affiliations:** ^1^ Key Laboratory of Genetics and Germplasm Innovation of Tropical Special Forest Trees and Ornamental Plants, Ministry of Education, College of Forestry, Hainan University, Haikou, Hainan, China; ^2^ College of Ecology and Environment, Hainan University, Haikou, Hainan, China; ^3^ Key Laboratory of Germplasm Resources Biology of Tropical Special Ornamental Plants of Hainan Province, College of Forestry, Hainan University, Haikou, China; ^4^ Tropical Crops Genetic Resources Institute, Chinese Academy of Tropical Agricultural Sciences, Haikou, Hainan, China

**Keywords:** fern gametophyte, light signal, photosynthesis, photoprotection, weighted gene co-expression network analysis

## Abstract

Light serves not only as a signaling cue perceived by plant photoreceptors but also as an essential energy source captured by chloroplasts. However, excessive light can impose stress on plants. Fern gametophytes possess the unique ability to survive independently and play a critical role in the alternation of generations. Due to their predominantly shaded distribution under canopies, light availability becomes a limiting factor for gametophyte survival, making it imperative to investigate their response to light. Previous research on fern gametophytes’ light response has been limited to the physiological level. In this study, we examined the light response of *Adiantum flabellulatum* gametophytes under different photosynthetic photon flux density (PPFD) levels and identified their high sensitivity to low light. We thereby determined optimal and stress-inducing light conditions. By employing transcriptome sequencing, weighted gene co-expression network analysis, and Gene Ontology and Kyoto Encyclopedia of Genes and Genomes analyses, we identified 10,995 differentially expressed genes (DEGs). Notably, 3 *PHYBs* and 5 Type 1 *CRYs* (*CRY1s*) were significantly down-regulated at low PPFD (0.1 μmol m^-2^ s^-1^). Furthermore, we annotated 927 DEGs to pathways related to photosynthesis and 210 to the flavonoid biosynthesis pathway involved in photoprotection. Additionally, we predicted 34 transcription factor families and identified a close correlation between *mTERFs* and photosynthesis, as well as a strong co-expression relationship between *MYBs* and *bHLHs* and genes encoding flavonoid synthesis enzymes. This comprehensive analysis enhances our understanding of the light response of fern gametophytes and provides novel insights into the mechanisms governing their responses to light.

## 1 Introduction

Ferns, an ancient lineage of land plants, have thrived on Earth for approximately 380 million years and are a fundamental component of the planet’s plant community (Schneider et al., 2004). Their gametophytes exhibit the remarkable ability to survive independently and serve as the site for the production and occurrence of sexual reproductive cells. Additionally, they play a critical role in the alternation of generations (also known as metagenesis) by nourishing the young sporophyte (Krieg and Chambers, 2022).

Light, a pivotal environmental factor for plant growth, serves both as a signal perceived by photoreceptors and as energy captured by chloroplasts to facilitate photosynthesis (de Wit et al., 2016; Poorter et al., 2019). However, excessive light can induce photostress, challenging photoprotective mechanisms (Takahashi and Badger, 2011). Fern gametophytes predominantly inhabit shaded habitats, where light conditions pose a limiting factor for their survival. Previous studies have revealed that gametophytes of *Trichomanes speciosum* can effectively conduct photosynthesis under weak light conditions, with 85% of the daytime light intensity being less than 1 μmol m^-2^ s^-1^ (equivalent to approximately 0.01% to 0.1% of full daylight exposure). Nevertheless, prolonged exposure to higher light levels (>50 μmol m^-2^ s^-1^) can lead to the demise of these gametophytes (Johnson et al., 2000). The growth and/or photosynthetic rates of gametophytes from *Onoclea sensibilis* and *Cibotium glaucum* exhibit an increasing trend within a certain range of light intensity, but excessive light inhibits their development (Miller and Miller, 1961; Friend, 1974). In contrast, fern gametophytes are adapted to lower light conditions than sporophytes (Nitta et al., 2021). However, research on the light response of fern gametophytes has thus far been confined to the physiological level.

Flavonoids are secondary metabolites that are widely present in bryophytes, pteridophytes (including lycophytes, horsetails, and ferns), and spermatophytes (Santos et al., 2017). These compounds have photoprotective properties and can absorb ultraviolet and some visible light, as well as function as antioxidants to scavenge reactive oxygen species generated by strong light exposure (Ferreyra et al., 2021). Previous studies have shown that environmental factors such as light can regulate the expression of flavonoid biosynthetic enzyme genes in the sporophytes of ferns. However, it is unknown whether the expression of flavonoid biosynthetic enzyme genes in the gametophytes of ferns is regulated by light. For example, research has found that the expression of flavonoid biosynthetic enzyme genes in fronds of *Azolla filiculoides*, such as *CHI*, *C4H*, *CHS*, and *DFR1*, is regulated by environmental factors such as light, which affects flavonoid synthesis (Costarelli et al., 2021). Similarly, studies on cultured spores of *Dryopteris fragrans* have shown that UV-B stress can increase the expression of the *DfCHS* gene and the total flavonoid content (Sun et al., 2014). Therefore, further research on the expression of flavonoid biosynthetic enzyme genes and their regulation by light in the gametophytes of ferns is of great significance for a deeper understanding of the photoprotective mechanism of fern gametophytes.

Transcription factors/transcriptional regulators are proteins that regulate gene expression (Strader et al., 2022). In the genomes of the model plant *Arabidopsis thaliana* and sorghum (*Sorghum bicolor*), the proportion of transcription factors/transcriptional regulators is 7.51% and 7.69%, respectively (Arabidopsis Genome, 2000; Tian et al., 2016). Despite their large genome size, the proportion of transcription factors/transcriptional regulators in ferns is relatively low (Sessa and Der, 2016). For example, in the transcriptomes of ferns *Huperzia serrata* and *Monachosorum maximowiczii*, the proportion of transcription factors/transcriptional regulators is only 1.87% and 1.11%, respectively (Liu et al., 2016; Yang et al., 2017). In the transcriptome of the gametophyte of *Adiantum flabellulatum* that we previously reported, the proportion of transcription factors/transcriptional regulators is 2.25% (Cai et al., 2022a). The genome of *Salvinia cucullata*, the smallest known genome among ferns, has only 983 transcription factors/transcriptional regulators, accounting for 4.94% of all genes, which is also lower than that in gymnosperms or angiosperms (Li et al., 2018). Currently, research on the regulatory role of transcription factors/transcriptional regulators in ferns is very limited, and we still do not understand how they respond to light, and whether there is a potential regulatory relationship with genes related to photoprotection.

*A. flabellulatum*, which typically grows under the canopy, is an acid-soil indicator plant (Liao et al., 2017) that has significant value in both ornamental and medicinal uses (Shahriar and Kabir, 2011). In this study, we used transcriptome sequencing, weighted gene co-expression network analysis, Gene Ontology (GO), Kyoto Encyclopedia of Genes and Genomes (KEGG) analysis, and real-time quantitative polymerase chain reaction (qPCR) to explore the genes responsive to light and potential regulatory relationships between transcription factors and genes related to flavonoid biosynthesis. Furthermore, by identifying key genes and pathways, this study enhances our understanding of the light response mechanism of *A. flabellulatum* gametophytes and provides a foundation for uncovering their molecular mechanism.

## 2 Materials and methods

### 2.1 Cultivation of *Adiantum flabellulatum* gametophytes

Spores of *A. flabellulatum* were collected from the rubber forest at Ma’an Mountain (109.519°E, 19.501°N) in Danzhou, Hainan Province, China, as described by Cai et al. (2022b). The spores were sterilized with 0.1% HgCl_2_ for 5 min, washed with sterile water five times, and then plated on 1/4 MS solid medium (3% w/v sucrose and 0.7% w/v agar). The plated spores were exposed to meticulously controlled illumination, with a photosynthetic photon flux density (PPFD) level of 7.1 μmol m^-2^ s^-1^, and maintained under an uninterrupted 24-hour photoperiod. Upon spore germination, young gametophytes with diameters ranging from 1 to 2 mm were transferred to fresh 1/4 MS solid medium, also containing 3% w/v sucrose and 0.7% w/v agar. Subsequently, the gametophytes were divided into seven distinct experimental groups, each cultivated under varying PPFD conditions: 0, 0.1, 1.4, 7.1, 14.4, 73.6, and 145.3 μmol m^-2^ s^-1^, respectively (**Supplementary Table 1**, **Supplementary Figure 1**). These cultivations were carried out over a continuous 40-day period under an uninterrupted 24-hour photoperiod. The perimeter and projected area of the gametophytes were quantified using Image J software (v1.8.0). Each treatment was replicated at least eight times, and the cultivation temperature was maintained at 25°C. Statistical analysis was conducted using the least significant difference (LSD) test.

### 2.2 RNA extraction, library construction and sequencing

The gametophytes of *A. flabellulatum* were cultivated under diverse light conditions: 0, 0.1, 7.1, and 145.3 μmol m^-2^ s^-1^, for a period of 12 days, maintaining an uninterrupted 24-hour photoperiod. Subsequently, the four sample groups, denoted as Af0, Af0.1, Af7.1, and Af145.3, were collected for transcriptome sequencing. Three biological replicates were established for each treatment, resulting in a total of 12 samples. Approximately 200 mg of gametophytes were collected from each sample, and total RNA was extracted using the CTAB method. mRNA was then enriched using oligo dT magnetic beads, fragmented, and reverse-transcribed into cDNA for library construction. Sequencing was performed on the DNBSEQ platform. The raw reads were filtered using SOAPnuke software (v1.5.2) to remove reads with adapters, N content exceeding 10%, and more than 50% of bases with a quality score below 15. The clean reads were aligned to the full-length transcriptome of *A. flabellulatum* gametophytes using Bowtie2 (v2.2.5), and gene expression levels were calculated using RSEM (v1.2.8). These steps were executed by the Beijing Genomics Institute (BGI). Finally, we conducted sequencing saturation analysis, correlation analysis, and principal component analysis using the method described by Cai et al. (2021).

### 2.3 Selection of differentially expressed genes and acquisition of co-expression gene modules

The study involved three comparison groups: Af0 vs Af0.1, Af0.1 vs Af7.1, and Af7.1 vs Af145.3. Genes with average expression levels less than 0.5 in each comparison group were removed, and differentially expressed genes (DEGs) were screened using the criteria of |log_2_ FoldChange| ≥ 1 and *Q*-Values < 0.001. The expression levels of all DEGs in the 12 samples were used to construct gene co-expression modules using the WGCNA package in R software, with “sft$powerEstimate=13” and “mergeCutHeight=0.25”. Modules that were strongly correlated with the phenotypes and light conditions of *A. flabellulatum* gametophyte were selected for further analysis.

### 2.4 GO and KEGG analysis

The module genes were annotated and classified using Blast2GO (v2.5.0) for Gene Ontology (GO, http://www.geneontology.org/) and Blastx (v2.2.23) and Diamond (v0.8.31) tools for KEGG (http://www.genome.jp/kegg/) annotation and classification, respectively (Cai et al., 2022b). Enrichment analysis was performed using the Phyper function package in the R software. BGI completed the GO and KEGG annotations. Enrichment pathways related to the gametophyte phenotype and light conditions of *A. flabellulatum* were selected for further analysis and discussion.

### 2.5 Prediction of transcription factor families

In this study, the identification of open reading frames (ORFs) from gene sequences was conducted using getorf (EMBOSS: 6.5.7.0). The resulting ORFs were aligned to transcription factor protein domains (http://plantregmap.gao-lab.org/) using hmmsearch (v3.0) (Tian et al., 2020). The genes’ potential for encoding transcription factors were then identified based on the transcription factor family characteristics outlined in PlantTFDB (http://planttfdb.gao-lab.org/) (Jin et al., 2017) and classified according to their respective transcription factor families.

### 2.6 Visualization of gene co-expression network

We used Cytoscape software (v3.9.1) to visualize the co-expression networks of flavonoid biosynthesis genes, as well as *MYBs* and *bHLHs* in the green, brown, yellow, and turquoise modules, following the method described by Shannon et al. (2003). In the visualization, nodes represent genes, and the size of each node is positively correlated with its degree. Lines represent the weight of co-expression between genes, with thicker and darker lines indicating higher weights.

### 2.7 Quantitative real-time polymerase chain reaction

Fifteen genes were chosen for validation by quantitative real-time polymerase chain reaction (qRT-PCR) (**Supplementary Table 2**). A total of 12 samples were used, with three technical replicates per sample. To achieve relative quantification, we selected isoform_78364 (*Elongation Factor Ts*), which exhibited stable expression across all samples, as the internal control gene, following the method described by Pfaffl (2001). Relative quantification was carried out using the 2^-ΔΔCt^ method, and significance analysis was performed using the LSD method.

## 3 Results

### 3.1 Effects of different PPFD levels on gametophyte growth of *Adiantum flabellulatum*

**Figure 1:**
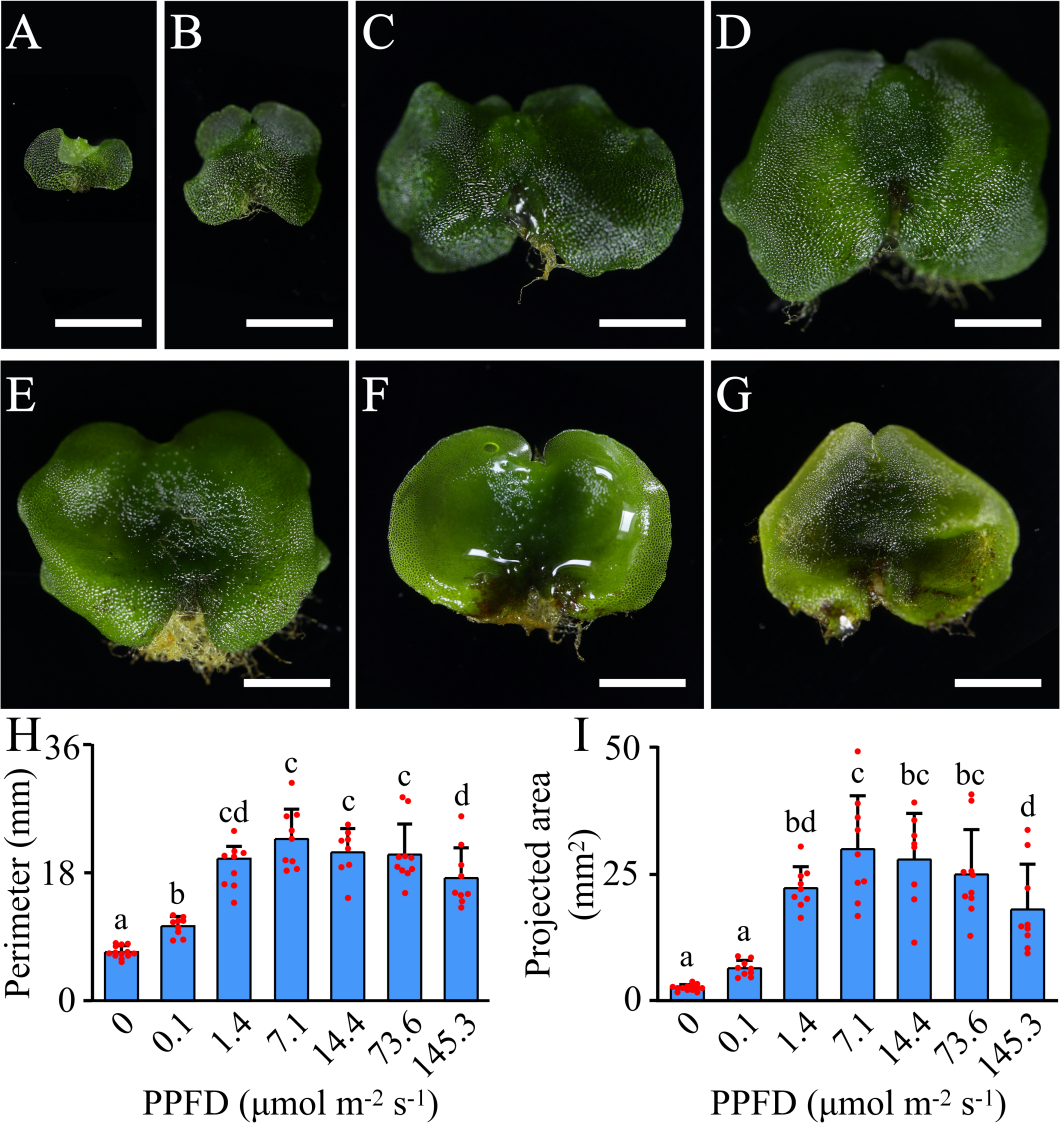
Effects of different PPFD levels on the growth of *A. flabellulatum* gametophytes. **(A–G)**: *A. flabellulatum* gametophytes were cultured for 40 days under PPFD levels of 0, 0.1, 1.4, 7.1, 14.4, 73.6, and 145.3 μmol m^-2^ s^-1^. **(H, I)**: The gametophyte perimeter **(H)** and projected area **(I)** were measured after 40 days of cultivation under different PPFD levels. Lowercase letters indicate significant differences (*p* < 0.05) between different PPFD levels. Scale bar = 2 mm.

The perimeters and projected areas of gametophytes grown under different PPFD levels were measured. The results showed that gametophytes are highly sensitive to low light, with their perimeter increasing significantly under a PPFD as low as 0.1 μmol m^-2^ s^-1^. Below 7.1 μmol m^-2^ s^-1^, the perimeter and/or projected area of gametophytes increased with increasing PPFD levels and reached a maximum at 7.1 μmol m^-2^ s^-1^. When the PPFD levels ranged between 7.1 μmol m^-2^ s^-1^ and 73.6 μmol m^-2^ s^-1^, the average value of the perimeter and projected area of gametophytes decreased slightly, but no significant differences were observed. However, when the PPFD reached 145.3 μmol m^-2^ s^-1^, the perimeter and projected area of gametophytes decreased significantly (**Figure 1**). Therefore, we conclude that 7.1 μmol m^-2^ s^-1^ is the optimal PPFD for the growth of *A. flabellulatum* gametophytes, while both PPFD levels below 7.1 μmol m^-2^ s^-1^ and at 145.3 μmol m^-2^ s^-1^ can induce light stress on the gametophytes.

### 3.2 Quality evaluation of transcriptome sequencing

We selected four treatments with PPFD levels of 0, 0.1, 7.1, and 145.3 μmol m^-2^ s^-1^ (represented by Af0, Af0.1, Af7.1, and Af145.3, respectively) to explore the genes involved in the response of *A. flabellulatum* gametophytes to light using transcriptome sequencing, based on previous experimental results. Three biological replicates were performed for each treatment, resulting in a total of 12 samples that were sequenced using the DNBSEQ platform. The full-length transcript sequences of *A. flabellulatum* gametophytes reported by Cai et al. (2022a) were used as reference genes for relative quantification.

**Figure 2:**
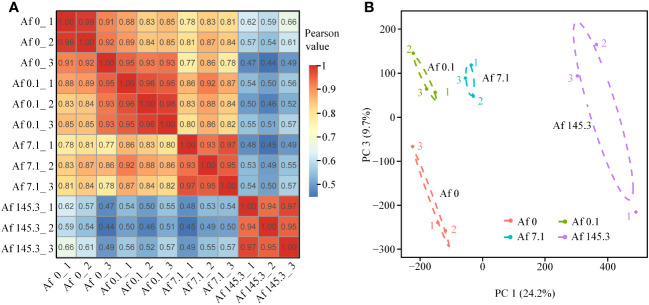
Pearson correlation coefficient analysis and principal component analysis of 12 samples. **(A, B)**: Pearson correlation coefficient analysis **(A)** and principal component analysis (PCA) **(B)** based on the expression data of all genes in 12 samples from the four treatments. The PCA results show that the four treatments are separated on a plane constructed by PC1 and PC3, indicating that these two principal components account for most of the variation among the treatments.

The raw read counts of each sample were greater than 42 M, and after filtering, each sample had at least 41 M clean reads, with a ratio greater than 96.17% and Q20 and Q30 values higher than 94% and 87%, respectively (**Supplementary Table 3**). Sequencing saturation analysis indicated that the gene identification ratio plateaued when the reads number exceeded 50×100 K, suggesting that the sequencing data had reached saturation (**Supplementary Figure 2A**). The gene expression level distributions of the 12 samples were analyzed, and the median gene expression levels were all ≥0.97 (**Supplementary Figure 2B**). Pearson correlation coefficients between biological replicates were ≥0.91 (**Figure 2A**), and the four treatments were separated from each other on the plane composed of PC1 and PC3 in the principal component analysis (**Figure 2B**). These results confirm the reliability and high quality of the sequencing data, which can be used for subsequent DEGs screening and analysis. The sequencing data have been deposited in the NCBI SRA database under accession number PRJNA868202.

### 3.3 Selection of differentially expressed genes

**Figure 3:**
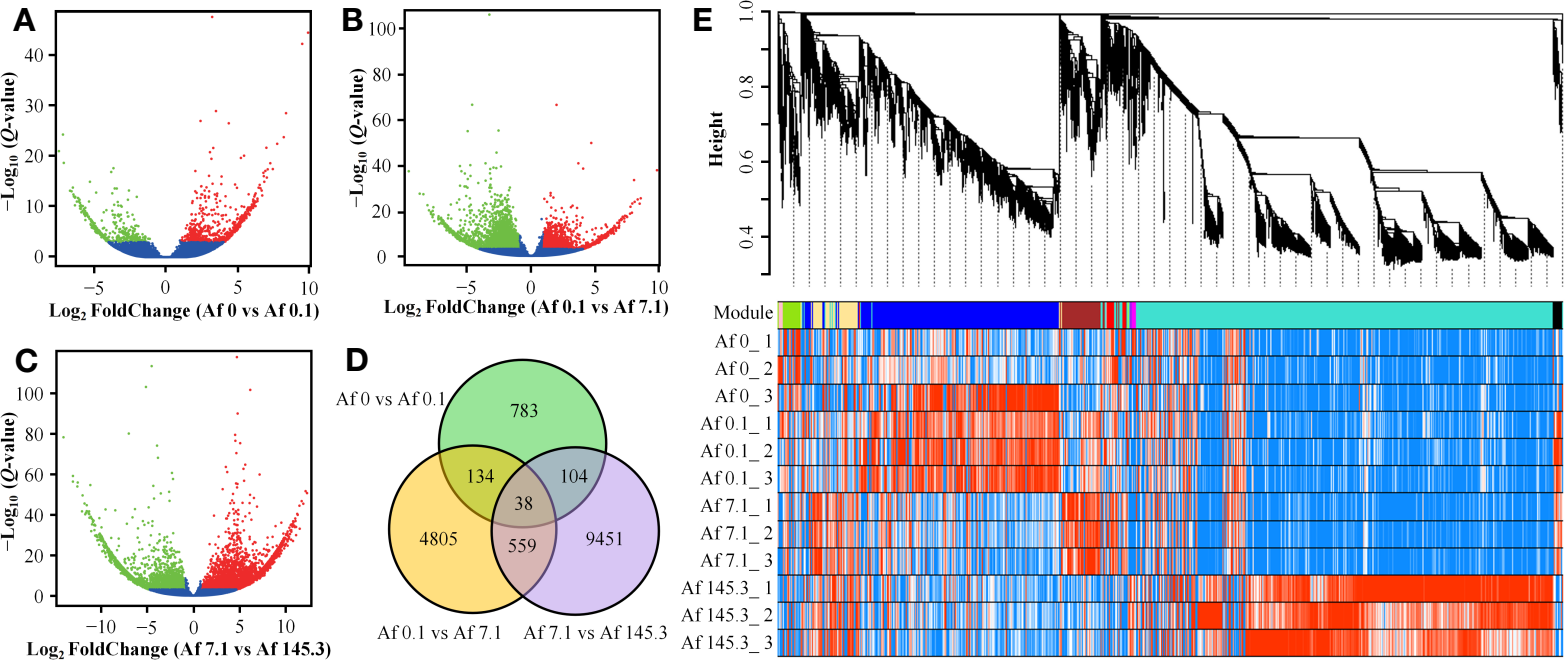
Identification of DEGs and weighted gene co-expression network analysis. **(A–C)**: DEGs were identified using criteria of |log_2_ FoldChange| ≥ 1 and *Q*-Values < 0.001. The red and green dots represent up-regulated and down-regulated DEGs in Af0 vs Af0.1 **(A)**, Af0.1 vs Af7.1 **(B)**, and Af7.1 vs Af145.3 **(C)**, respectively. **(D)**: Venn diagram of DEGs distribution among three comparison groups, with the numbers in overlapping regions representing the shared DEGs. **(E)**: Dendrogram and gene modules of a total of 15,874 DEGs obtained through hierarchical clustering analysis. The turquoise module contains the largest number of DEGs.

A total of 248,028 genes were detected among the 12 samples. Three comparison groups were set: Af0 vs Af0.1, Af0.1 vs Af7.1, and Af7.1 vs Af145.3. In each comparison group, we filtered out genes with an average expression level of less than 0.5 for both treatments and then selected DEGs with |log_2_ FoldChange| ≥ 1 and *Q*-Values < 0.001. This resulted in 1,059, 5,536, and 10,152 DEGs, respectively. The Af7.1 vs Af145.3 comparison had the most DEGs, with 7,692 upregulated and 2,460 downregulated genes, followed by Af0.1 vs Af7.1, which had 1,864 upregulated and 3,672 downregulated genes. The Af0 vs Af0.1 comparison had the fewest DEGs (**Supplementary Tables 4**–**6**; **Figures 3A–C**). After removing duplicates, a total of 15,874 DEGs were obtained (**Figure 3D**).

For these 15,874 DEGs, we performed weighted gene co-expression analysis and obtained 12 gene co-expression modules (**Figure 3E**). The turquoise module contained 8,796 DEGs and was positively correlated with “PPFD levels,” while the yellow (746 DEGs) and brown (875 DEGs) modules were positively correlated with “gametophyte area.” The green module (387 DEGs) was positively correlated with “presence or absence of light” (**Supplementary Table 7**). In contrast, the black module (191 DEGs) was negatively correlated with “presence or absence of light” (**Supplementary Table 7**, **Supplementary Figure 3**). Based on these results, we selected DEGs from these five modules for further analysis.

### 3.4 Gene ontology analysis of module genes

**Figure 4:**
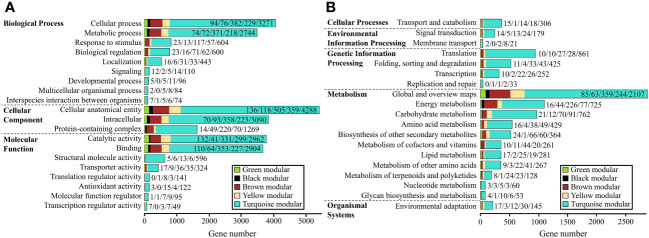
GO and KEGG classifications of the five gene modules. **(A, B)**: GO **(A)** and KEGG **(B)** classifications of genes in the five modules. The numbers on each bar represent the count of genes for the green, black, brown, yellow, and turquoise modules, respectively.

We performed GO annotation on a total of 10,995 DEGs from the five gene modules mentioned earlier. Of these, 8,033 were successfully annotated, accounting for 73.06% of the total. These annotations covered three major functional categories: “Biological process,” “Cellular component,” and “Molecular function,” which contained 4,911, 5,451, and 6,424 genes, respectively. In the “Biological process” category, all five modules had the highest number of genes annotated in the “cellular process” term, while in the “Cellular component” category, it was the “cellular anatomical entity.” In the “Molecular function” category, the black and brown modules had the highest number of genes annotated in the “binding” term, while the green, yellow, and turquoise modules were most enriched in the “catalytic activity” term (**Supplementary Tables 8**, **9**; **Figure 4A**).

We present the top 21 enriched GO terms with *Q*-values < 0.01. Firstly, the green module was enriched in at least 8 terms related to light signaling (8/21). Among them, “photoreceptor activity” had the smallest *Q*-value (*Q*=1.67E-07) and the most genes (8) (**Supplementary Table 10**, **Supplementary Figure 4A**). This indicates that the DEGs in the green module play an important role in regulating light signaling during the development of *A. flabellulatum*’s gametophyte. Secondly, most of the terms in the black and brown modules were related to photosynthesis. For example, “thylakoid” had the smallest *Q*-value (*Q*=1.63E-28) and the most genes (52) in the black module, while “regulation of photosynthesis, dark reaction” had the highest enrichment rate (0.11) (**Supplementary Table 10**, **Supplementary Figure 4B**). In the brown module, “photosystem II” had the smallest *Q*-value (*Q*=2.35E-115) and the most genes (166), while “photosynthetic acclimation” had the highest enrichment rate (0.125) (**Supplementary Table 10**, **Supplementary Figure 4C**). This indicates that the DEGs in the black and brown modules mainly participate in regulating photosynthesis during the development of *A. flabellulatum*’s gametophytes. Additionally, the two terms with the smallest *Q*-value in the yellow module were “flavonoid biosynthetic process” (*Q*=3.61E-09) and “flavonoid metabolic process” (*Q*=3.87E-09), and they both had the most genes (12) (**Supplementary Table 10**, **Supplementary Figure 4D**). The terms enriched in the turquoise module were mainly related to cell damage, such as “cell killing” and “killing of cells of other organism,” and they had the most genes (36) (**Supplementary Table 10**, **Supplementary Figure 4E**). This indicates that the DEGs in the yellow and turquoise modules mainly participate in resisting light stress caused by strong light.

### 3.5 KEGG analysis of module genes

We performed KEGG annotation on 10,995 DEGs, of which 5,405 genes were successfully annotated with an annotation rate of 49.16%. These genes were classified into five major categories, including “Cellular processes”, “Environmental information processing”, “Genetic Information Processing”, “Metabolism”, and “Organismal Systems”. Among these categories, the “Metabolism” category had the most DEGs, with a total of 3,363 genes. The five gene modules were annotated to the most genes in the “Transport and catabolism” (354 genes) of “Cellular processes”, “Signal transduction” (235 genes) of “Environmental information processing”, “Global and overview maps” (2,858 genes) of “Metabolism”, and “Environmental adaptation” (207 genes) of “Organismal Systems”. In the “Genetic Information Processing” category, the black and turquoise modules had the most genes annotated in “Translation”, while the green, brown, and yellow modules were involved in “Folding, sorting and degradation”. Importantly, in the “Metabolism” category, all five gene modules had a relatively high number of genes annotated in “Energy metabolism” (1,088 genes) and “Carbohydrate metabolism” (956 genes), indicating that the regulation of these two metabolisms by light is also crucial (**Supplementary Tables 11**, **12**; **Figure 4B**).

**Figure 5:**
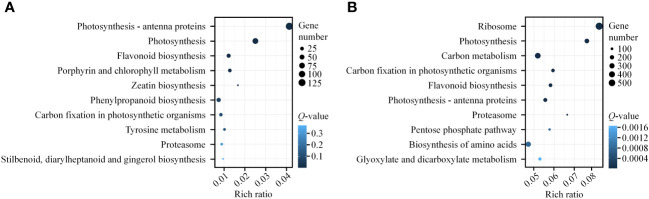
KEGG pathway enrichment analysis of brown and turquoise modules. **(A, B)**: KEGG pathway enrichment analysis of genes in the brown **(A)** and turquoise **(B)** modules. The top 10 enriched pathways with the smallest *Q*-values are presented. The size of the circle corresponds to the number of genes in the pathway, and the color intensity reflects the level of significance (darker colors indicate smaller *Q*-values).

We presented the top 10 KEGG pathways with the smallest *Q*-values in enrichment analysis (**Supplementary Figure 5**; **Figure 5**). Firstly, the green, black, and yellow gene modules were all enriched in the “Circadian rhythm-plant” pathway, with the green module having the smallest *Q*-value and the largest number of genes (14) in this pathway (**Supplementary Table 13**, **Supplementary Figure 5A**). Secondly, the black, yellow, brown, and turquoise gene modules were all enriched in multiple pathways related to photosynthesis, such as “Photosynthesis-antenna proteins” and “Photosynthesis” (**Supplementary Table 13**). The black module had the smallest *Q*-value (1.63E-20), highest enrichment rate (0.009240924), and the largest number of genes (28) in the “Photosynthesis” pathway (**Supplementary Table 13**, **Supplementary Figure 5B**). In the brown module, the “Photosynthesis-antenna proteins” pathway had the smallest *Q*-value (1.08E-77), highest enrichment rate (0.041363336), and the largest number of genes (125) (**Supplementary Table 13**; **Figure 5A**). Additionally, the brown, yellow, and turquoise gene modules were enriched in the “Flavonoid biosynthesis” pathway, which is involved in photoprotection (**Supplementary Table 13**, **Supplementary Figure 5C**; **Figure 5**). In summary, our KEGG analysis results also indicate that light plays an essential regulatory role in light signal transduction, photosynthesis, and photoprotection during gametophyte development in *A. flabellulatum*.

### 3.6 Analysis of transcription factor families for module genes

We predicted transcription factor families for the 10,995 DEGs and identified 34 families, with mTERF, MYB, and bHLH families having the highest number of DEGs at 13 each. The turquoise module had the most predicted transcription factor families (30) and individual transcription factors (95), followed by the yellow module with 13 families and 17 individual factors (**Supplementary Table 14**, **Supplementary Figure 6**).

### 3.7 Expression analysis of photosynthesis-related genes

**Figure 6:**
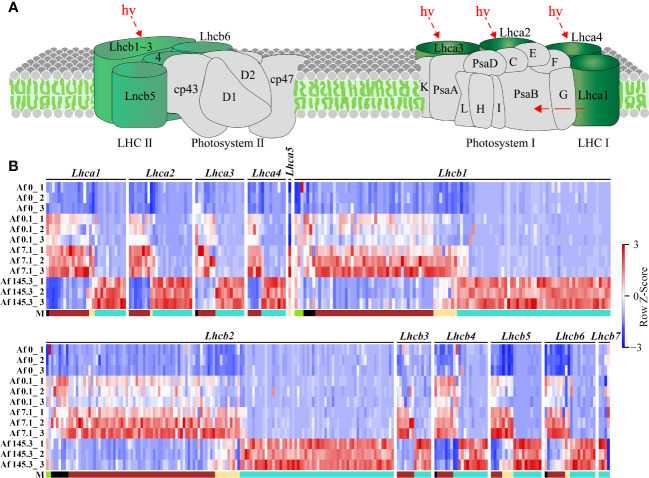
Expression of DEGs in the “Photosynthesis-antenna proteins” pathway across 12 samples. **(A)**: Diagram of the “Photosynthesis-antenna proteins” pathway (source: https://www.kegg.jp/pathway/map00196, accessed on April 14, 2023); **(B)**: Expression profiles of DEGs encoding 12 subunits of LHCI and LHCII across 12 samples. “M” represents the gene module. It can be observed that there are more DEGs in the brown and turquoise modules for this pathway.

**Figure 7:**
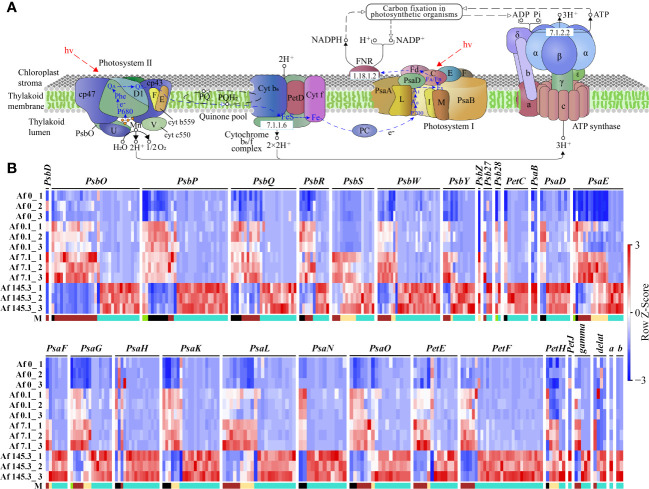
Expression of DEGs in the “Photosynthesis” pathway across 12 samples. **(A)**: Diagram of the “Photosynthesis” pathway (source: https://www.kegg.jp/entry/map00195, accessed on April 14, 2023); **(B)**: Expression profiles of DEGs encoding 30 subunits of the PSII complex (11 subunits: PsbD, PsbO, PsbP, PsbQ, PsbR, PsbS, PsbW, PsbY, PsbZ, Psb27, Psb28), cytochrome b_6_/f complex (1 subunit: PetC), PSI complex (10 subunits: PsaB, PsaD, PsaE, PsaF, PsaG, PsaH, PsaK, PsaL, PsaN, PsaO), photosynthetic electron transfer chain (4 subunits: PetE, PetF, PetH, PetJ), and ATP synthase (4 subunits: gamma, delta, a, b) in 12 samples. “M” represents gene modules. The brown and turquoise modules contain a larger number of genes.

As the “Photosynthesis-antenna proteins” and “Photosynthesis” pathways are significantly enriched in multiple gene modules, we first analyzed the expression of DEGs in these two pathways. The “Photosynthesis-antenna proteins” pathway includes Light-harvesting complex I (LHC I) and Light-harvesting complex II (LHC II), with LHC I containing five subunits (Lhca1-Lhca5) and LHC II containing seven subunits (Lhcb1-Lhcb7). Results showed that among the five gene modules, the “Photosynthesis-antenna proteins” pathway had a total of 327 DEGs, including the 12 subunits mentioned above, mainly belonging to the brown (125) and turquoise (168) modules. These genes were generally expressed at the highest levels under a PPFD of 7.1 μmol m^-2^ s^-1^ (brown module) or 145.3 μmol m^-2^ s^-1^ (turquoise module) (**Supplementary Table 15**; **Figure 6**). In addition, the “Photosynthesis” pathway includes the Photosystem II complex (PSII complex), cytochrome b_6_/f complex, Photosystem I complex (PSI complex), photosynthetic electron transfer chain, and Adenosine triphosphate synthase (ATP synthase). Results showed that among the five gene modules, the “Photosynthesis” pathway had a total of 370 DEGs, including 30 subunits, such as 11 subunits of the PSII complex (PsbD, PsbO, PsbP, PsbQ, PsbR, PsbS, PsbW, PsbY, PsbZ, Psb27, Psb28), one subunit of the cytochrome b_6_/f complex (PetC), 10 subunits of the PSI complex (PsaB, PsaD, PsaE, PsaF, PsaG, PsaH, PsaK, PsaL, PsaN, PsaO), 4 subunits of the photosynthetic electron transfer chain (PetE, PetF, PetH, PetJ), and 4 subunits of ATP synthase (gamma, delta, a, b). These DEGs were also mainly distributed in the brown (76) and turquoise (231) modules (**Supplementary Table 16**, **Figure 7**).

We also analyzed the DEGs involved in Calvin cycle (141), chlorophyll (61), and carotenoid (28) biosynthesis, and found that these DEGs were mainly classified into the turquoise module (**Supplementary Tables 17**-**19**, **Supplementary Figures 7**-**9**).

Moreover, mitochondrial transcription termination factors (mTERFs) located in plastids play important regulatory roles in the development and function of chloroplasts. *AfmTERFs* were detected in the brown, yellow, and turquoise modules, with 1, 1, and 11 members, respectively. Therefore, we constructed a phylogenetic tree of mTERFs from *A. flabellulatum* and *Arabidopsis*. The results showed that four AfmTERFs (isoform_43473, isoform_47623, isoform_50012, and isoform_240354) in *A. flabellulatum* were homologous to AtmTERF6, one (isoform_20996) was homologous to AtmTERF9, and two (isoform_83065 and isoform_91611) were homologous to AtmTERF12 (**Supplementary Table 20**, **Supplementary Figure 10**). Interestingly, all seven *AfmTERFs* belonged to the turquoise module.

### 3.8 Expression of flavonoid biosynthesis genes

**Figure 8:**
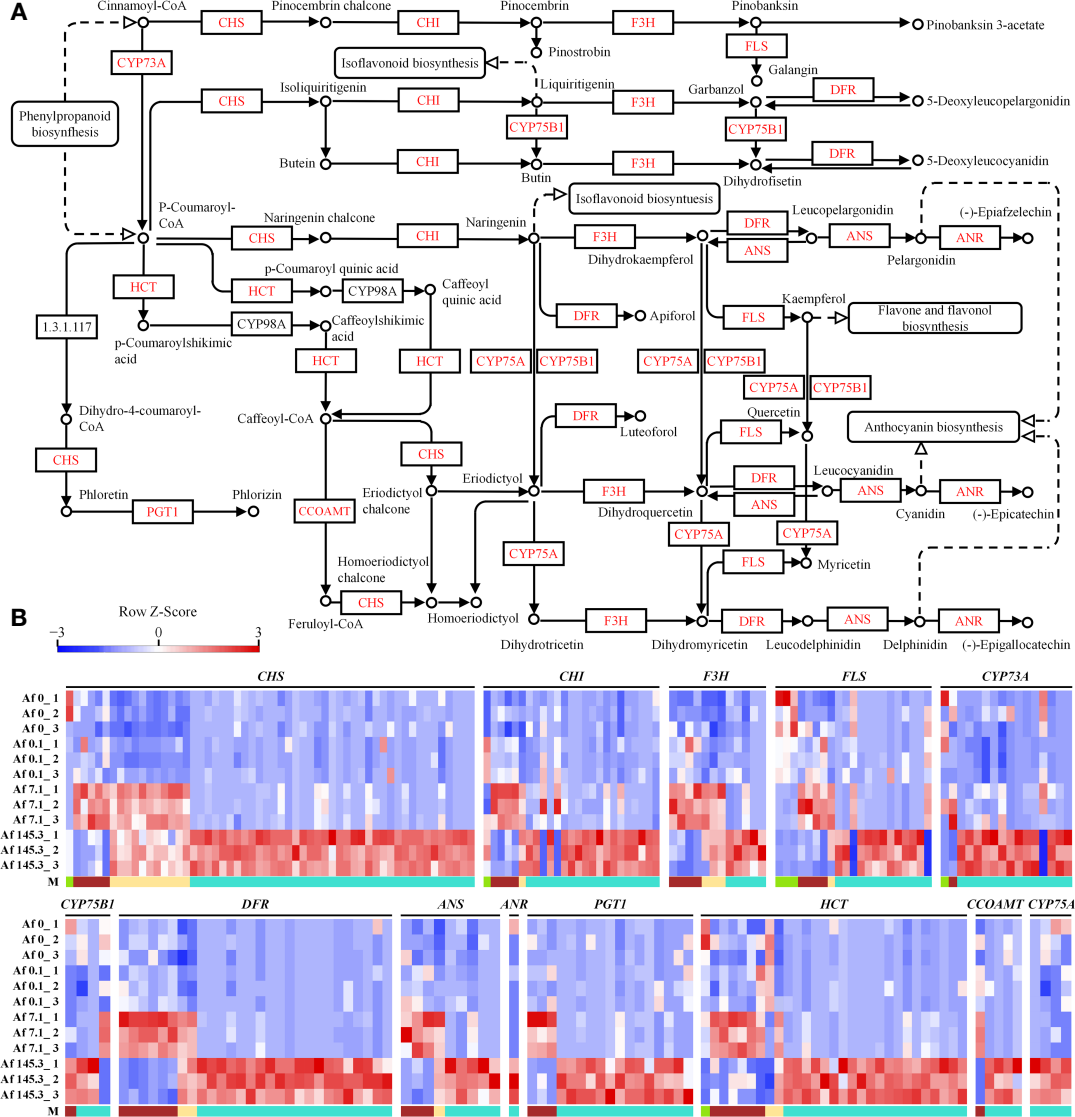
Expression of DEGs in the “Flavonoid Biosynthesis” pathway across 12 samples. **(A)**: “Flavonoid Biosynthesis” pathway (Source: https://www.kegg.jp/pathway/map00941, accessed on April 14, 2023); **(B)**: Expression profiles of DEGs encoding CHS, CHI, F3H, FLS, CYP73A, CYP75B1, DFR, ANS, ANR, PGT1, HCT, CCOAMT, and CYP75A in 12 samples. “M” represents gene modules, with a higher number of DEGs observed in the turquoise module.

Based on the analysis in the previous sections, we found that the flavonoid biosynthesis pathway is significantly enriched in multiple gene modules, particularly in the brown, yellow, and turquoise modules. Therefore, we analyzed the expression of DEGs in this pathway. Our results show that there are 210 DEGs encoding 13 enzymes, including CHS, CHI, F3H, and FLS, which are key enzymes in the flavonoid biosynthesis pathway. These DEGs mainly belong to the turquoise module (**Supplementary Table 21**; **Figure 8**). Thus, we suggest that light, especially strong light, strongly upregulates the expression of genes encoding flavonoid biosynthesis enzymes in the gametophyte of *A. flabellulatum*.

**Figure 9:**
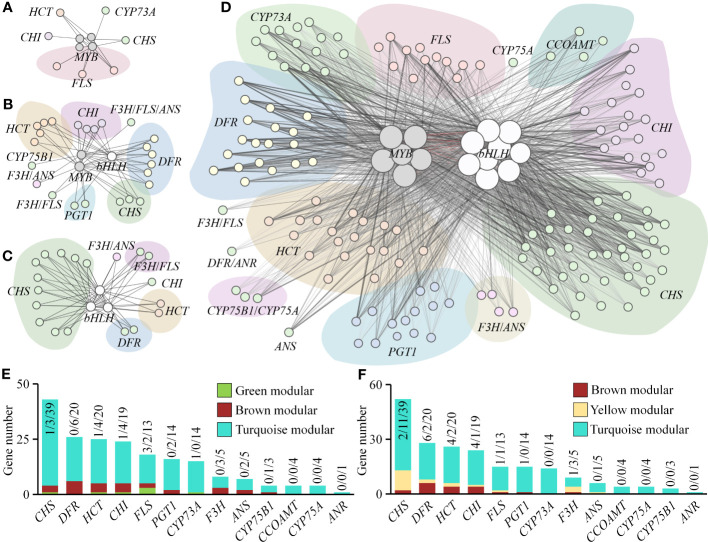
Co-expression network of flavonoid biosynthesis genes and MYB/bHLH transcription factor genes. **(A–D)**: Co-expression networks of flavonoid biosynthesis genes and MYB/bHLH transcription factor genes in green, brown, yellow, and turquoise modules, respectively. Nodes represent genes, with larger nodes indicating higher degree values. Lines represent co-expression weights between genes, with thicker and darker lines indicating higher weight values. **(E, F)**: Number of co-expressed flavonoid biosynthetic enzyme-coding genes with MYB and bHLH transcription factor genes, respectively. Additionally, we found a co-expression relationship between *AfMYBs* and *AfbHLHs* in the turquoise module, indicated by red lines.

The MYB and bHLH families of transcription factors are important in plant responses to abiotic stress. These two types of transcription factors were detected in four modules, with the highest number found in the turquoise module. Therefore, we performed co-expression analysis of flavonoid biosynthesis genes and MYB/bHLH transcription factor genes in *A. flabellulatum*. Our results showed that the encoding genes of flavonoid biosynthesis enzymes, CHS, DFR, HCT, and CHI, have a wide range of co-expression relationships with *MYBs* and *bHLHs*, with 39, 20, 20, and 19 genes in the turquoise module, respectively (**Supplementary Table 22**; **Figure 9**). Furthermore, we also found co-expression relationships between *MYBs* and *bHLHs* in the turquoise module.

### 3.9 Quantitative real-time polymerase chain reaction

**Figure 10:**
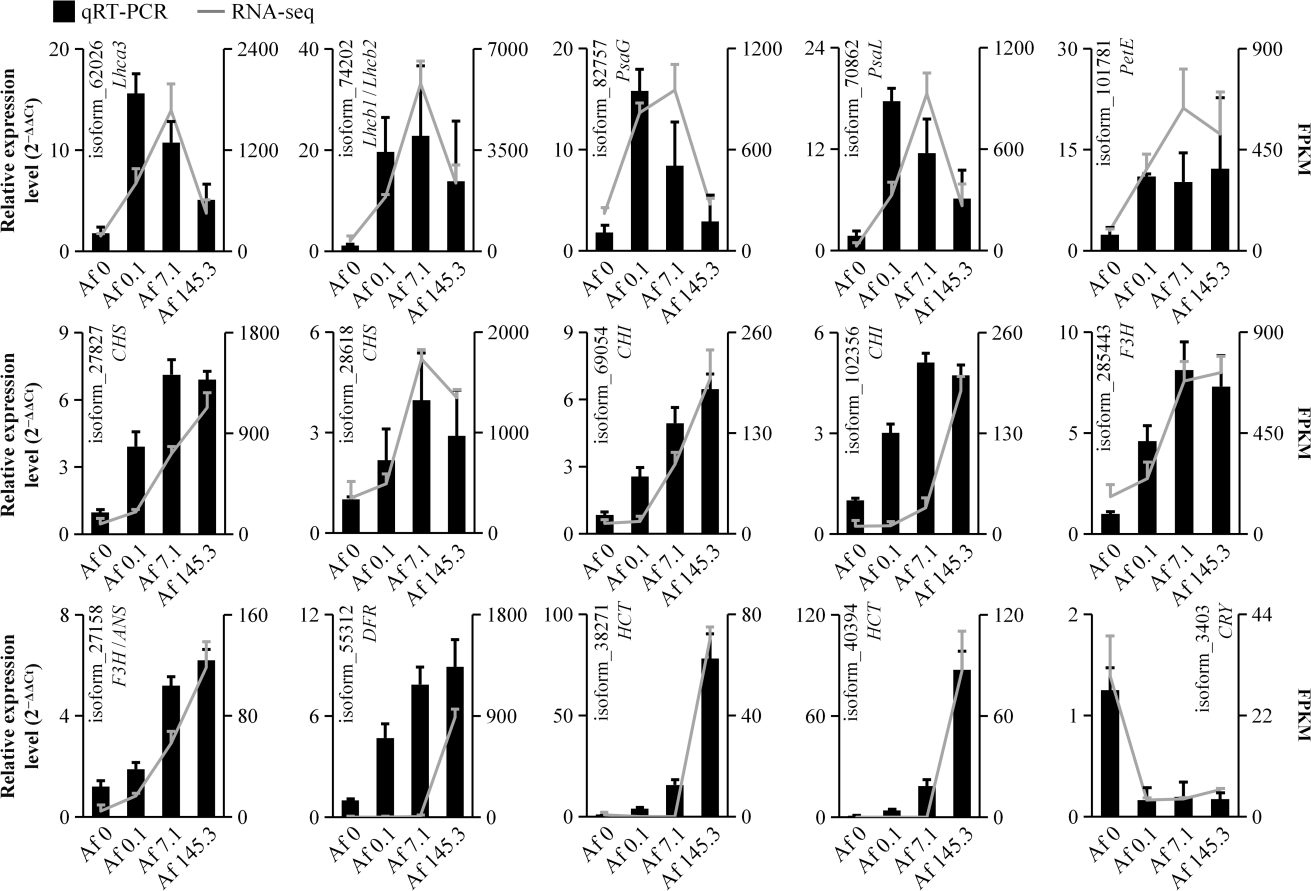
Real-time quantitative polymerase chain reaction. The expression trends of 15 DEGs were validated by qRT-PCR, with the bar chart and line chart representing the results of qRT-PCR and transcriptome, respectively. The consistency between qRT-PCR and transcriptome results suggests the reliability of the transcriptome data.

To validate the accuracy of the transcriptomic data, we selected 15 DEGs for qRT-PCR analysis. These included two genes from the “Photosynthesis-Antenna Proteins” pathway (*Lhca3* and *Lhcb1*/*Lhcb2*), three genes from the “Photosynthesis” pathway (*PsaG*, *PsaL*, and *PetE*), nine genes from the “Flavonoid Biosynthesis” pathway (*CHSs*, *CHIs*, *F3H*, *ANS*, *DFR*, and *HCTs*), and *Cryptochrome* (*CRY*). Our qRT-PCR results demonstrated that the expression patterns of these 15 genes were consistent with the transcriptomic data, confirming the reliability of our transcriptomic analysis (**Supplementary Table 23**; **Figure 10**).

## 4 Discussion

The gametophytes of ferns are small in size and need to constantly sense and utilize the scattered sunlight that passes through the forest canopy to survive. In our study, GO enrichment analysis of the green module identified 8 out of 21 enriched terms related to light signaling. Notably, “photoreceptor activity” had the smallest *Q*-value and the highest number of genes (**Supplementary Figure 4A**). Additionally, KEGG enrichment analysis showed significant enrichment of the “Circadian rhythm-plant” pathway in the green module, with the smallest *Q*-value and a relatively large number of genes (**Supplementary Figure 5A**). Phytochromes (PHYs) and cryptochromes (CRYs) are the primary photoreceptors that mediate light-induced signaling and regulate circadian rhythms in plants (Guo et al., 1998; Oakenfull and Davis, 2017). In the green module, we identified three *PHYB* genes and five *CRY1* genes that were significantly downregulated at a PPFD of only 0.1 μmol m^-2^ s^-1^ (**Supplementary Table 24**, **Supplementary Figure 11**). We also observed a significant increase in gametophyte perimeter under a low PPFD of 0.1 μmol m^-2^ s^-1^. As *A. flabellulatum* predominantly thrives in humid understory environments (Yang, 2015), we conclude that its gametophytes exhibit high sensitivity to low light, a trait likely linked to their adaptation to such conditions.

Mitochondrial transcription termination factors (mTERFs) are a class of transcriptional regulators encoded by nuclear genes. Originally discovered in animal mitochondria, mTERFs have been found to play a role in transcriptional regulation not only in animal mitochondria but also in plant chloroplasts and mitochondria (Roberti et al., 2009; Babiychuk et al., 2011). In *Arabidopsis*, AtmTERF6 is localized to both chloroplasts and mitochondria, while AtmTERF9 and AtmTERF12 are localized to chloroplasts. Among them, AtmTERF6 and AtmTERF9 have been shown to play important roles in photosynthesis and chloroplast development (Robles et al., 2015; Romani et al., 2015; Xu et al., 2017; Zhang et al., 2018), while the function of AtmTERF12 remains relatively limited (Xu et al., 2017). Single mutants of *mterf6* and *mterf9* in *Arabidopsis* exhibited reduced levels of 16S and 23S rRNA of the small and large plastid ribosomal subunits in chloroplasts, decreased number of chloroplast ribosomes, and affected protein translation in chloroplasts, leading to impaired chloroplast development, reduced chlorophyll a and b content, decreased number of mesophyll cells, and leaf whitening (Robles et al., 2015; Romani et al., 2015; Robles et al., 2018; Zhang et al., 2018; Meteignier et al., 2021). In comparison, the double mutant *mterf6-5 mterf9* in *Arabidopsis* seedlings exhibited a more pronounced pale/albino phenotype, indicating a synergistic effect between the genes (Robles et al., 2018). In this study, we discovered that there are five AfmTERFs (isoform_47623, isoform_43473, isoform_20996, isoform_83065, and isoform_91611) in the gametophytes of *A. flabellulatum*, which show homology to AtmTERF6, AtmTERF9, or AtmTERF12, and their encoding genes were up-regulated under strong light (145.3 μmol m^-2^ s^-1^). Given the localization and functional significance of mTERFs in other plant systems, we speculate that these AfmTERFs may play a crucial role in balancing the effects of light damage in fern gametophytes under strong light stimulation, ensuring the safe and efficient photosynthetic process. Further studies should aim to elucidate the precise molecular mechanisms underlying AfmTERF function and their impact on fern gametophyte development and survival.

Flavonoids play a vital role in photoprotection in plants by scavenging free radicals and suppressing the generation of reactive oxygen species (Agati et al., 2012). Strong light exposure promotes the expression of genes encoding flavonoid biosynthesis enzymes, such as CHS, CHI, and F3H, in various angiosperms, including *Arabidopsis thaliana*, *Petunia*, and *Medicago sativa* seedlings, enhancing flavonoid biosynthesis and providing photodamage defense. In this study, we found that light exposure, particularly strong light, significantly increased the expression of genes encoding 13 flavonoid biosynthesis enzymes in *A. flabellulatum* gametophytes, including key enzymes such as CHS, CHI, and F3H. This is the first report of such induction in fern gametophytes, although it has been reported in various angiosperms (Albert et al., 2009; Gao et al., 2019; Zheng et al., 2021). Meanwhile, we detected stable total flavonoid content in *A. flabellulatum* gametophytes under different PPFD levels (**Supplementary Figure 12**). Therefore, we hypothesize that the upregulation of relevant enzyme gene expression maintains the stable total flavonoid content during the process of responding to photodamage in *A. flabellulatum* gametophytes.

Transcription factors, particularly MYB and bHLH, regulate the expression of flavonoid biosynthesis genes. Previous studies have demonstrated that many MYB transcription factors can regulate the expression of flavonoid biosynthesis genes such as *CHS*, *CHI*, *F3H*, and *DFR*, promoting flavonoid biosynthesis (Dubos et al., 2010). For instance, AtMYB11, AtMYB12, and AtMYB111 in *Arabidopsis* can directly bind to the promoter of flavonoid biosynthesis genes like *AtCHS*, *AtCHI*, and *AtF3H*, directly regulating the expression of flavonoid biosynthesis genes (Stracke et al., 2007). Additionally, MYB can form binary or ternary complexes with bHLH and WD40 to co-regulate the expression of flavonoid biosynthesis genes (Xu et al., 2015), as seen in the MYB-bHLH-WD40 ternary complex in *Arabidopsis* and the MYB10-bHLH3 binary protein complex in red-fleshed apples (*Malus sieversii* f. *niedzwetzkyana*), which directly regulate the expression of *DFR*, promoting anthocyanin biosynthesis (Gonzalez et al., 2016; Wang et al., 2017). Light closely regulates *MYB* expression, as seen with *MdMYB1* in apples (*Malus domestica*) and *PpMYB10* in red Chinese sand pears (*Pyrus pyrifolia*), which are induced by light and regulate the expression of *DFR*, *CHS*, and *ANS* genes, promoting flavonoid biosynthesis (Takos et al., 2006; Bai et al., 2017). In this study, we found that several MYB and bHLH transcription factors in *A. flabellulatum* have a strong co-expression relationship with flavonoid biosynthesis genes encoding CHS, CHI, DFR, and F3H. Therefore, we hypothesize that under strong light irradiation, MYB and bHLH transcription factors in *A. flabellulatum* gametophytes are activated to regulate the expression of flavonoid biosynthesis genes, promoting flavonoid biosynthesis and playing a photoprotective role.

In summary, this study not only provides valuable insights into the protection and cultivation of *A. flabellulatum* but also serves as a reference for further research on gametophytes of ferns’ responses to light. The comprehensive analysis of the light response in *A. flabellulatum* gametophytes sheds light on their sensitivity to different light conditions, which is likely an adaptation to their natural understory habitats. Furthermore, the identification of *AfmTERFs* and their potential role in photoprotection enhances our understanding of fern gametophyte survival strategies under varying light intensities. The upregulation of flavonoid biosynthesis genes under strong light exposure highlights the significance of these secondary metabolites in photoprotection, offering potential avenues for future research on fern photobiology and stress responses.

## Data availability statement

The datasets presented in this study can be found in online repositories. The names of the repository/repositories and accession number(s) can be found below: https://www.ncbi.nlm.nih.gov/genbank/, PRJNA868202.

## Author contributions

ZC and JL conceived and designed the study. ZC, XW and ZX performed experiments. ZC, XW, ZX and ZW analyzed the data. ZC, XW, ZX, ZW, XS and JL drafted the manuscript. ZC, XW, ZX, XY, SX and JL finalized the manuscript. JL, ZC and XY provided the funding acquisition. All authors contributed to the article and approved the submitted version.
