# Supplementary material for: Light response of gametophyte in Adiantum flabellulatum: transcriptome analysis and identification of key genes and pathways
Source: Front Plant Sci. 2023 Sep 7;14:1222414. doi: 10.3389/fpls.2023.1222414 (PMC10513451; doi:10.3389/fpls.2023.1222414)
Supplement: Supplementary file 1 [file DataSheet_1.pdf]

Legends to Supplementary Figures 1-12

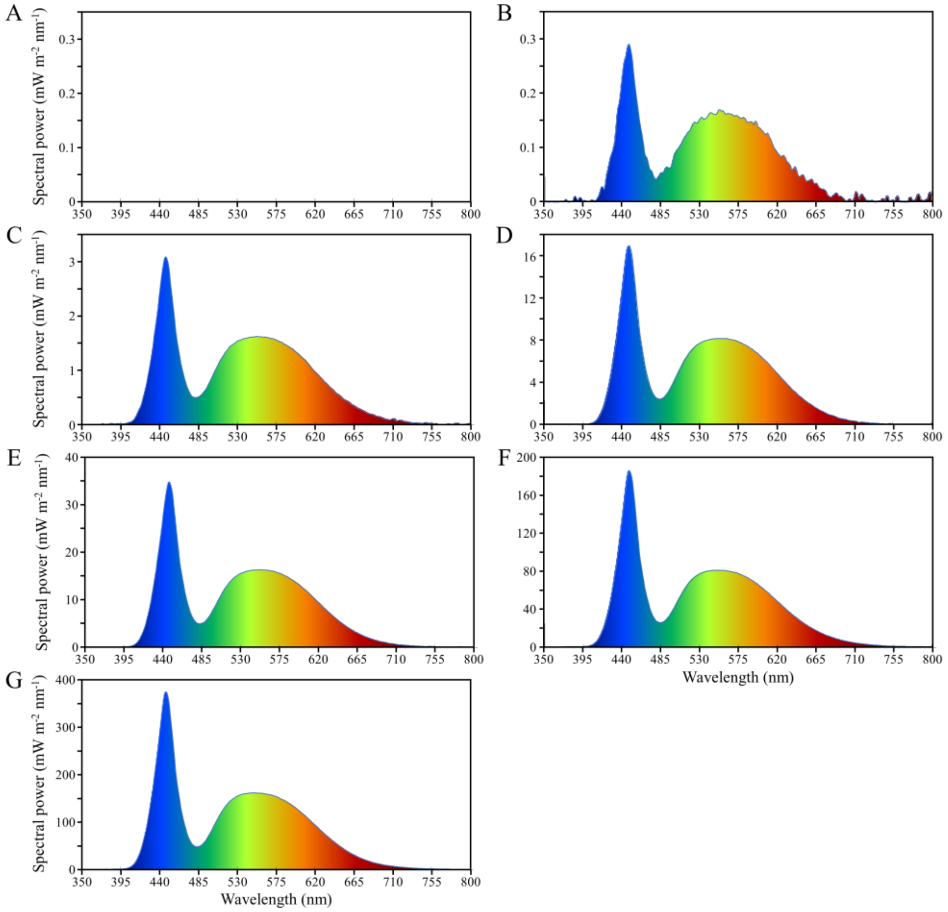

Supplementary Figure 1. Light spectra at 7 different photosynthetic photon flux density (PPFD) levels

(**A**)-(**G**): The light spectra of white light sources at PPFD levels of 0 (**A**), 0.1 (**B**), 1.4 (**C**), 7.1 (**D**), 14.4 (**E**), 73.6 (**F**), and 145.3 (**G**) μmol m^{-2} s^{-1}, respectively.

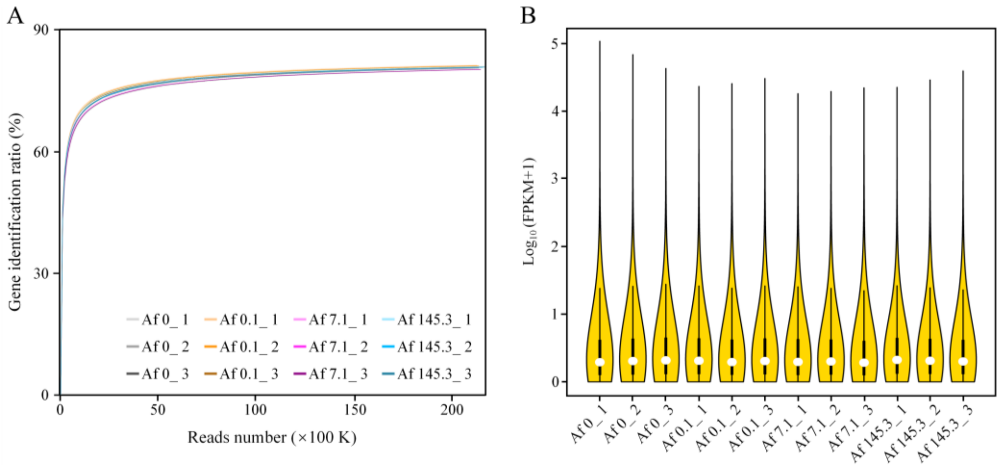

Supplementary Figure 2. Sequencing saturation and gene expression distribution analysis of 12 samples

(**A**): Sequencing saturation analysis of 12 samples. The gene identification rate tended to plateau when the reads number exceeded 50×100 K, indicating that the sequencing data reached saturation. (**B**): Gene expression distribution of 12 samples. The outer contour represents the density distribution of genes (the wider the contour, the greater the gene density), the black box in the middle represents the quartile range, the white dot represents the median, and the thin black line represents the 95% confidence interval.

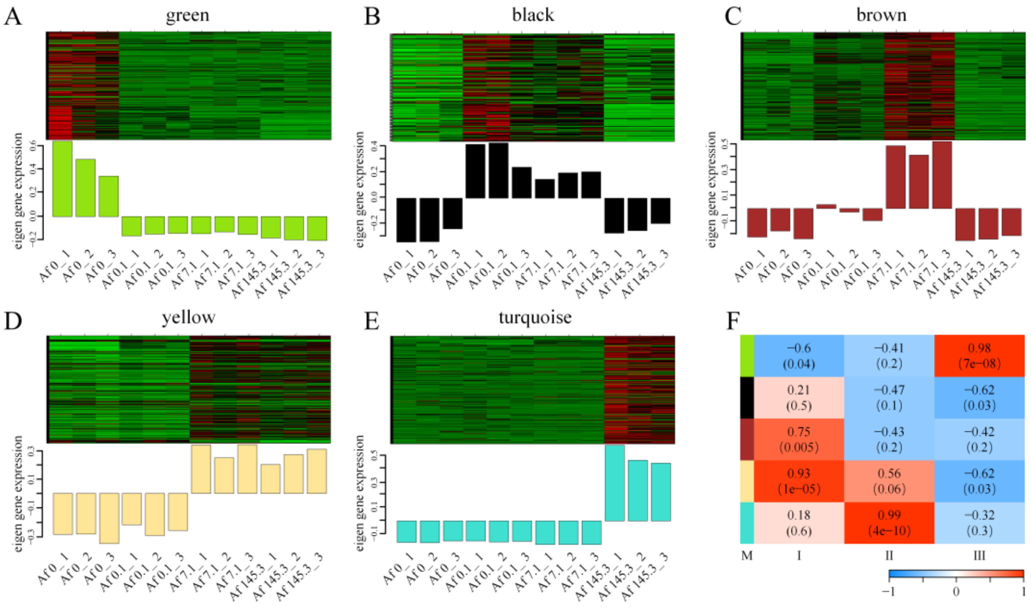

Supplementary Figure 3. Information presentation of five gene modules

(**A**)-(**E**): Heatmaps and eigen gene expression of differentially expressed genes (DEGs) in green (**A**), black (**B**), brown (**C**), yellow (**D**), and turquoise (**E**) gene modules across 12 samples. (**F**): Correlation heatmap between each gene module and the "gametophyte area", "PPFD levels", and "presence/absence of light". "M" denotes the module, and "I-III" denote "gametophyte area", "PPFD levels", and "presence/absence of light", respectively. Numbers outside and inside the parentheses represent correlation coefficients and *P*-values, respectively.

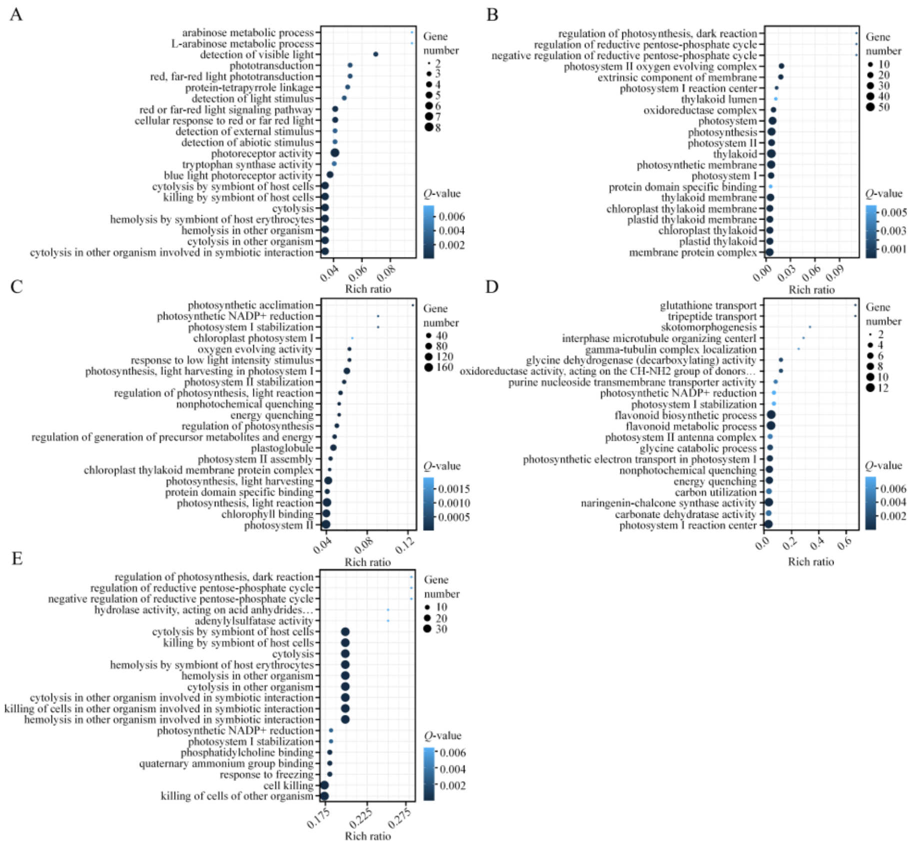

Supplementary Figure 4. GO enrichment analysis of genes in the five modules

(**A**)-(**E**): GO enrichment analysis of genes in the green (**A**), black (**B**), brown (**C**), yellow (**D**), and turquoise (**E**) modules. The top 21 enriched GO terms with *Q*-value < 0.01 are shown, where the size of the circle represents the number of genes, and darker colors indicate smaller *Q*-values.

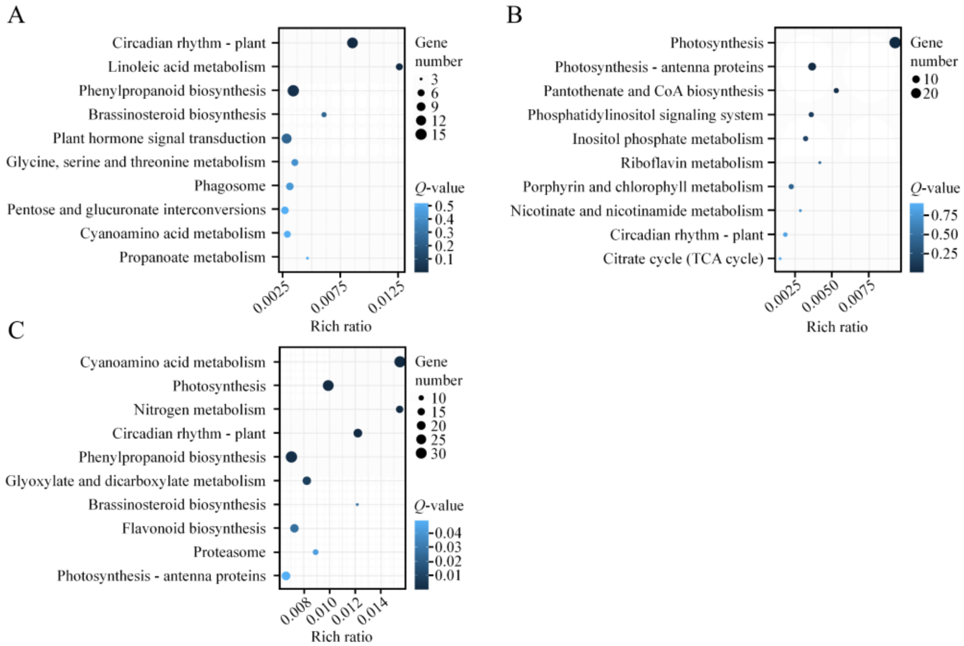

Supplementary Figure 5. KEGG pathway enrichment analysis of genes in the green, black, and yellow modules

(**A**)-(**C**): KEGG pathway enrichment analysis of genes in the green (**A**), black (**B**), and yellow (**C**) modules. The top 10 enriched pathways with the smallest *Q*-values are displayed, where the size of the circle represents the number of genes in the pathway and the color depth indicates the level of significance (the darker the color, the smaller the *Q*-value).

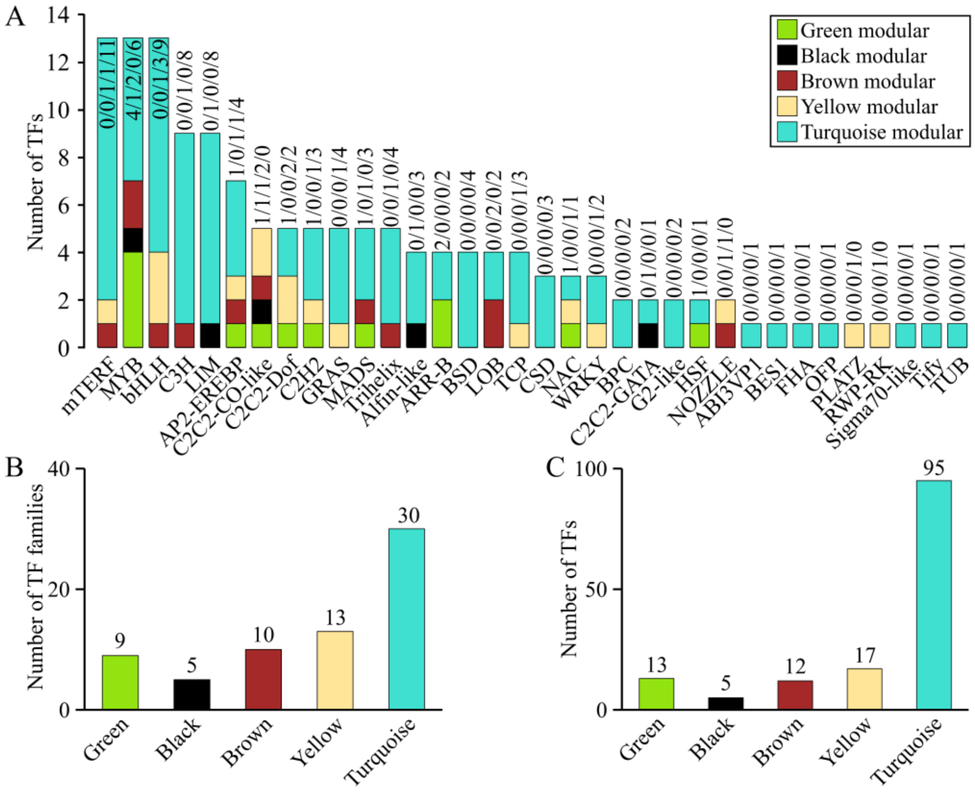

Supplementary Figure 6. Transcription factor prediction for genes in five modules

(**A**): Number of predicted transcription factor families in the five gene modules, with numbers from bottom to top representing the number of predicted transcription factors in the green, black, brown, yellow, and turquoise modules. (**B**)-(**C**): Number of predicted transcription factor families (**B**) and total number of predicted transcription factors (**C**) in the five gene modules.

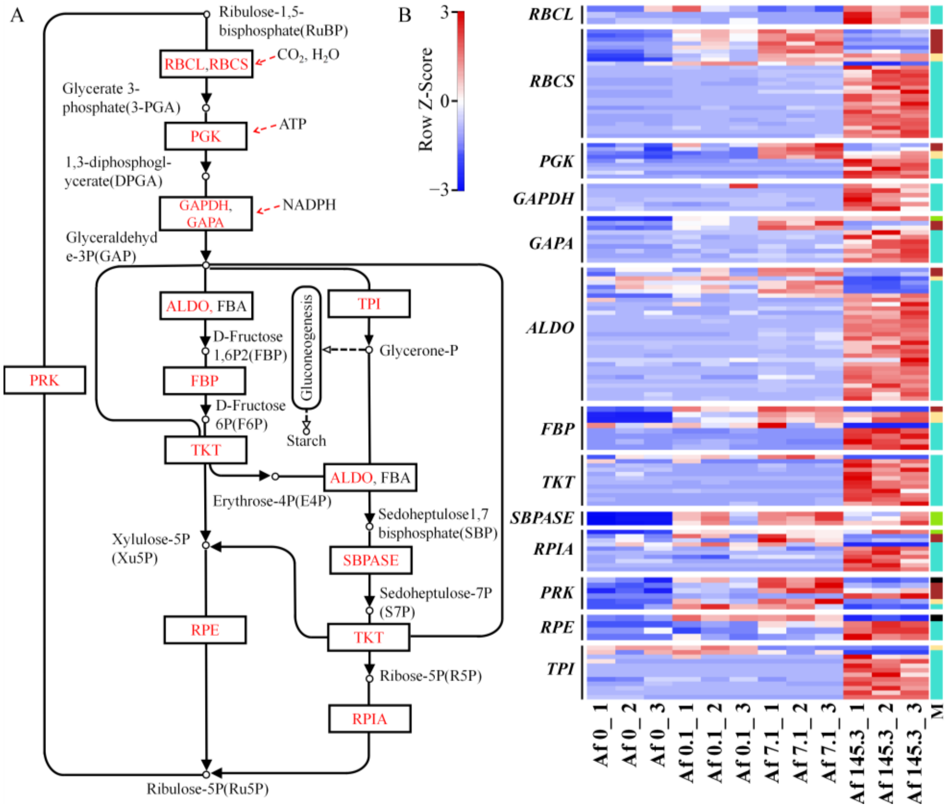

Supplementary Figure 7. Expression of DEGs in the "Calvin cycle" pathway across 12 samples

(**A**): Diagram of the "Calvin cycle" pathway (source: https://www.kegg.jp/entry/map00710, accessed on April 14, 2023); (**B**): Expression profiles of DEGs encoding RBCL, RBCS, PGK, GAPDH, GAPA, ALDO, FBP, TKT, SBPASE, RPIA, PRK, RPE and TPI in 12 samples. "M" represents gene modules, and it can be observed that there are more DEGs in the turquoise module.

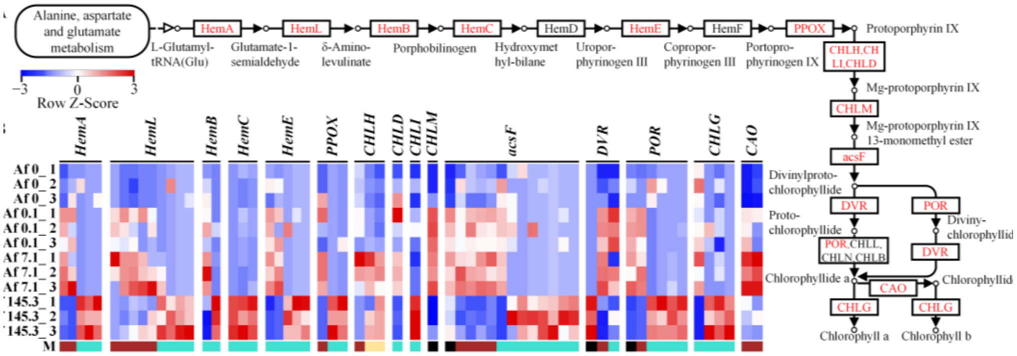

Supplementary Figure 8. Expression of DEGs in the "Chlorophyll Biosynthesis" pathway across 12 samples

(**A**): Diagram of the "Chlorophyll Biosynthesis" pathway (source: https://www.kegg.jp/entry/map00860, accessed on April 14, 2023); (**B**): Expression profiles of DEGs encoding HemA, HemL, HemB, HemC, HemE, PPOX, CHLH, CHLD, CHLI, CHLM, acsF, DVR, POR, CHLG, and CAO in 12 samples. "M" represents gene modules, and it is observed that the brown and turquoise modules have more DEGs.

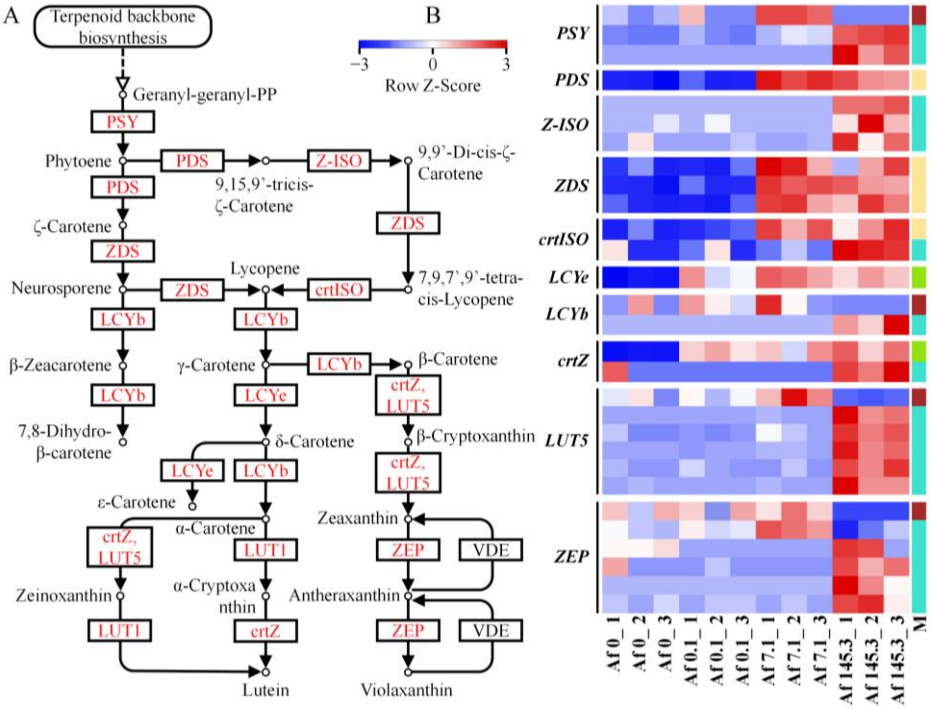

Supplementary Figure 9. Expression of DEGs in the "Carotenoid Biosynthesis" pathway across 12 samples

(**A**): Diagram of the "Carotenoid Biosynthesis" pathway (source: https://www.kegg.jp/entry/map00906, accessed on August 17, 2022). (**B**): Expression profiles of DEGs encoding PSY, PDS, Z-ISO, ZDS, crtISO, LCYe, LCYb, crtZ, LUT5, and ZEP in 12 samples. "M" represents gene modules, and it can be observed that the turquoise module has a relatively high number of DEGs.

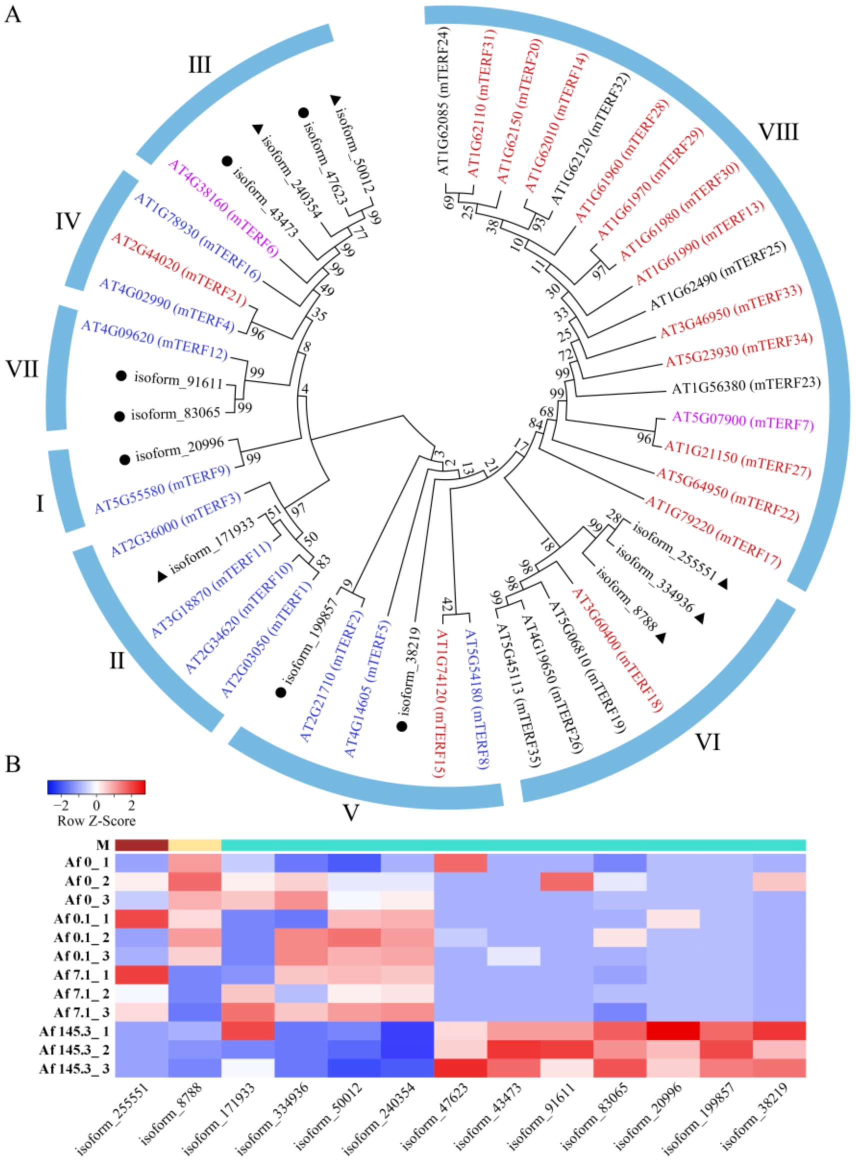

Supplementary Figure 10. Phylogenetic tree of mTERFs and their gene expression across 12 samples

(**A**): The phylogenetic tree of mTERFs was constructed using the Neighbor-Joining method based on the mTERF protein sequences of *A. flabellulatum* and *Arabidopsis thaliana*. The mTERFs were divided into eight groups. The blue and red fonts represent mTERFs localized in chloroplasts and mitochondria, respectively, while the purple font indicates mTERFs that can be found in both chloroplasts and mitochondria (Babiychuka et al., 2011; Zhang et al., 2018). (**B**): Heatmap showing the expression levels of *AfmTERFs* in 12 samples. The letter "M" denotes the gene module, and it is observed that there are more genes in the turquoise module.

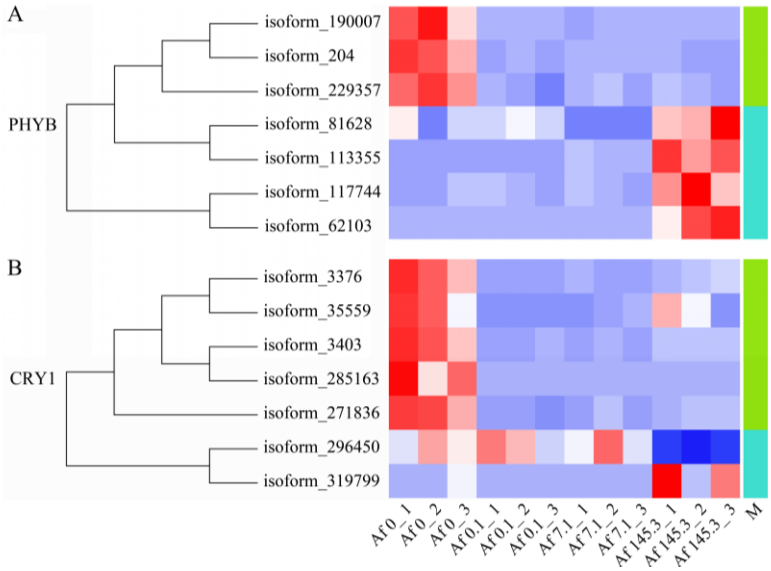

Supplementary Figure 11. Expression of two photoreceptor coding genes in 12 samples of the gametophyte of *A. flabellulatum*

(**A**)-(**B**): The expression profiles of 7 *AfPHYB* and 7 *AfCRY1* genes selected from five gene modules in 12 samples are shown. "M" indicates the module to which the gene belongs. The protein sequences were clustered and the evolutionary tree was drawn using the Neighbor-Joining (NJ) method.

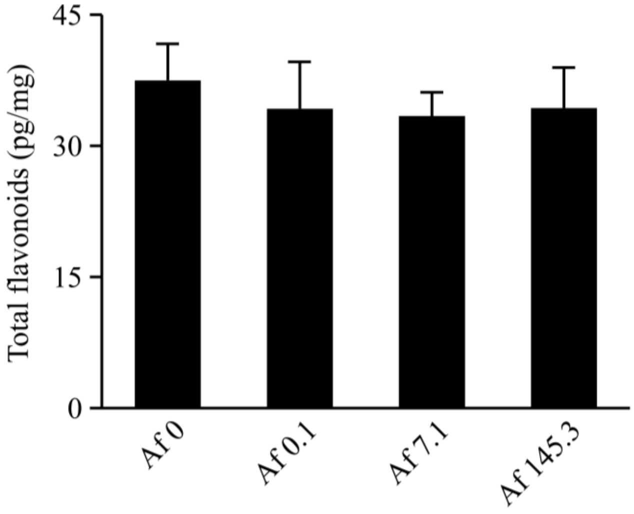

Supplementary Figure 12. Total flavonoid content of *A. flabellulatum* gametophyte

Total flavonoid content of *A. flabellulatum* gametophyte cultured for 12 days under different PPFD levels (0, 0.1, 7.1, and 145.3 μmol m^{-2} s^{-1}).
