# Supplementary material for: Light response of gametophyte in Adiantum flabellulatum: transcriptome analysis and identification of key genes and pathways
Source: Front Plant Sci. 2023 Sep 7;14:1222414. doi: 10.3389/fpls.2023.1222414 (PMC10513451; doi:10.3389/fpls.2023.1222414)
Supplement: Supplementary file 2 [file DataSheet_2.pdf]

Supplementary Tables 1-24 guidance

Supplementary Table 1: Seven parameters of light conditions for *Adiantum flabellulatum* gametophyte cultivation

Supplementary Table 2: Information of primers used in qRT-PCR

Supplementary Table 3: Summary of transcriptome data for the 12 samples

Supplementary Table 4: 1,059 DEGs in the Af 0 vs Af 0.1 comparison group

Supplementary Table 5: 5,536 DEGs in the Af 0.1 vs Af 7.1 comparison group

Supplementary Table 6: 10,152 DEGs in the Af 7.1 vs Af 145.3 comparison group

Supplementary Table 7: Correlation between 12 gene modules and "gametophyte area", "PPFD levels", "presence/absence of light"

Supplementary Table 8: GO annotations of the green, black, brown, yellow, and turquoise module genes

Supplementary Table 9: GO classification statistics of genes in the five modules, green, black, brown, yellow, and turquoise

Supplementary Table 10: GO enrichment of genes in five modules, green, black, brown, yellow, and turquoise

Supplementary Table 11: KEGG annotations of the green, black, brown, yellow, and turquoise module genes

Supplementary Table 12: KEGG pathway classification statistics of genes in the five modules, green, black, brown, yellow, and turquoise

Supplementary Table 13: KEGG pathway enrichment of genes in five modules, green, black, brown, yellow, and turquoise

Supplementary Table 14: Annotation of transcription factors in the five modules of green, black, brown, yellow, and turquoise

Supplementary Table 15: The expression levels of DEGs involved in photosynthesis - antenna proteins pathway

Supplementary Table 16: The expression levels of DEGs involved in photosynthesis pathway

Supplementary Table 17: The expression levels of DEGs involved in Calvin cycle

Supplementary Table 18: The expression levels of DEGs involved in chlorophyll biosynthesis

Supplementary Table 19: The expression levels of DEGs involved in Carotenoids biosynthesis

Supplementary Table 20: Protein sequences of 13 AfmTERFs

Supplementary Table 21: The expression levels of DEGs involved in flavonoid biosynthesis pathway

Supplementary Table 22: Co-expression network of flavonoid biosynthesis genes and MYB/bHLH transcription factor genes

Supplementary Table 23: Processing and analysis of qRT-PCR data

Supplementary Table 24: Expression profiles of 7 *AfPHYB* and 7 *AfCRY1* genes in 12 samples
